# Comparative associations of body mass index and waist-to-height ratio with cardiometabolic and functional health risk markers in children aged 6–12 years - the Health Oriented Pedagogical Project (HOPP)

**DOI:** 10.1371/journal.pone.0347538

**Published:** 2026-05-08

**Authors:** Per Morten Fredriksen, Morten Lindberg, Nandu Goswami, Asgeir Mamen

**Affiliations:** 1 University of Inland Norway, Faculty of Social and Health Sciences, Norway; 2 Vestfold Hospital Trust, Central Laboratory, Norway; 3 Gravitational Physiology and Medicine Research Unit, Division of Physiology and Pathophysiology, Otto Loewi Research Center, Medical University of Graz, Austria; 4 Center for Space and Aviation Medicine, College of Medicine, Mohammed Bin Rashid University of Medicine and Health Sciences, Dubai; 5 Kristiania University College, School of Health Sciences, Norway; Niigata University, JAPAN

## Abstract

**Background:**

Body mass index (BMI) and waist-to-height ratio (WHtR) are widely used markers of adiposity in children, but their relative associations with cardiometabolic and functional health measures remain debated.

**Methods:**

We analyzed data from children aged 6–12 years (n = 2297) in the Health Oriented Pedagogical Project (HOPP) with annual assessments from 2015–2020. Associations between BMI or WHtR and outcomes (body composition, blood lipids, blood pressure, physical activity, and cardiorespiratory fitness/endurance) were examined using linear mixed-effects models with repeated measures (random intercept for child; study year as the repeated factor). BMI and WHtR were evaluated in separate models.

**Results:**

Both BMI and WHtR were consistently associated with less favorable profiles across outcomes, including higher adiposity, lower fitness/endurance and MVPA, higher blood pressure, and a more adverse lipid profile (lower HDL and higher non-HDL cholesterol). Across most outcomes, differences between BMI- and WHtR-based models were modest; however, WHtR generally showed slightly stronger associations for lipid measures and fitness-related outcomes.

**Conclusions:**

BMI and WHtR demonstrate largely comparable associations with cardiometabolic and functional health measures in children aged 6–12 years. WHtR may provide complementary information on central adiposity and can be considered alongside BMI in pediatric screening and surveillance.

## Introduction

Childhood obesity has become a critical global health challenge and recent estimates indicate that more than 340 million children and adolescents aged 5–19 were overweight or obese [1–3]. Excess adiposity in childhood is associated with a host of adverse cardiometabolic outcomes, including insulin resistance, dyslipidemia, and elevated blood pressure, and strongly predicts the persistence of obesity and elevated cardiovascular risk into adulthood [1–3]. Beyond cardiometabolic complications, obesity can impair children’s functional health, for example by reducing cardiorespiratory fitness and physical mobility, and it often coexists with lower physical activity levels [4]. Early identification of children at risk is therefore paramount for prevention, motivating the use of simple anthropometric indices to screen for potential health impacts of excess weight.

### Anthropometric indices for childhood obesity

A variety of anthropometric measures are used to assess obesity in children, with body mass index (BMI) being the most ubiquitous. BMI (weight/height²) provides a convenient proxy for overall adiposity and is the basis for standard definitions of childhood overweight and obesity (typically using age- and sex-specific percentiles (isoBMI) or z-scores) [5,6]. BMI’s popularity stems from its simplicity and extensive validation in relation to health outcomes. However, it is well recognized that BMI cannot distinguish body fat from lean mass and may misclassify muscular or large-bodied children as overweight [5]. Moreover, BMI does not capture fat distribution; individuals with the same BMI can have very different central vs. peripheral fat patterns [5,7]. This limitation is important because central (abdominal) obesity is most strongly linked to cardiometabolic risk [5,7].

Waist circumference (WC) is often used alongside BMI to gauge central adiposity, but WC must be interpreted relative to body size (height) and lacks universal pediatric cutoffs. The waist-to-height ratio (WHtR) has emerged as a convenient index of central fat that accounts for stature [5,7–10]. WHtR (WC/height) and a simple universal threshold of 0.5 has been widely proposed for both children and adults. Notably, this criterion appears to be applicable across sexes, age groups, and ethnicities [5]. However, a recent meta-analysis of 41 studies found that the ideal threshold may vary by ethnic group (e.g., WHtR ≥ 0.46 in East/Southeast Asian youth and ≥0.54 in Latin American youth maximized detection of cardiometabolic risk) [11].

It has been argued that WHtR may better identify individuals with high visceral adiposity who are at risk despite a normal BMI, thereby capturing “early health risk” missed by BMI alone [5,7–10]. One analysis suggested that relying on BMI alone would misclassify about 10% of the UK population who actually have increased cardiometabolic risk [5]. Because of such considerations, clinical and public health guidelines have started to incorporate WHtR. UK National Institute for Health and Care Excellence (NICE) in 2022 recommended that children and adolescents (aged ≥5 years) consider monitoring their WHtR to better assess central obesity-related health risks [12]. WHtR can serve as an indicator of intra-abdominal fat accumulation and associated conditions (type 2 diabetes, hypertension, cardiovascular disease) across diverse populations.

### BMI vs. WHtR

Overall, BMI and WHtR perform similarly in identifying children at elevated cardiometabolic risk, although WHtR offers some practical advantages. Two earlier meta analyses reported on BMI, WHtR, and waist circumference, showing moderate for detecting outcomes of hypertension, high blood glucose, adverse lipid profiles, and metabolic syndrome in youth [13–15]. WHtR did *not* exhibit significantly better discriminatory power than BMI or WC for most outcomes, however, WHtR was slightly superior for identifying high triglycerides compared to BMI, and for identifying a high composite metabolic risk score compared to WC [13–15]. Despite the lack superiority in predictive power, WHtR’s simplicity of measurement and interpretation using a single cutoff for children and adults alike, makes it an attractive screening tool in practice [13]. Sardinha et al. (2016) analyzed data on over 4,200 children and adolescents and found that BMI, WC, and WHtR were all similarly associated with a clustered cardiometabolic risk score, comprising blood pressure, lipids, and insulin resistance markers [16]. The magnitudes of association did not differ appreciably between the three indices, and their ability to discriminate “at-risk” youths was likewise similar (AUC ~ 0.70–0.74 for each) [16]. Zong et al. (2024) reinforces this parity using data from 34,224 children across 14 countries, finding no significant differences between WHtR, BMI, and WC in detecting youths with multiple cardiometabolic risk factors (AUCs 0.77–0.78) [14,15]. The authors concluded that BMI and WHtR are *equally effective* for flagging children with cardiometabolic risk, and suggested WHtR could be used as a convenient alternative in clinical screening given its ease of use [14,15].

In terms of body composition, BMI is well correlated with total body fat percentage in children, although it cannot distinguish fat from lean tissue. Interestingly, a study reported that BMI explained a greater proportion of the variance in DXA-measured body fat percentage than WHtR did (R² ≈ 0.68 for BMI vs 0.50 for WHtR) in overweight/obese children [17]. These data underscore that BMI, despite its limitations, is a reasonably strong proxy for total adiposity in children, whereas WHtR specifically captures central fat. In theory, WHtR could better identify children with disproportionately high abdominal fat who are at cardiometabolic risk even if their weight is only moderately elevated. In practice, however, the literature to date suggests that both indices correlate strongly with key metabolic risk factors in youth [17]. In a cohort of overweight young children, BMI, WC, and WHtR all showed positive correlations with systolic blood pressure, HOMA-IR, leptin, and triglycerides, and no single measure was consistently superior for all parameters [17]. WHtR had a slightly higher correlation with triglycerides, whereas BMI was slightly more correlated with blood pressure and insulin resistance, but these differences were not large [17]. Likewise, a study found that a low WHtR (<0.5) was strongly associated with a favorable *cholesterol profile* and other metabolic health indicators in 10-year-old children [18]. Also, having higher fitness was independently associated with better HDL, whereas having WHtR < 0.5 was associated with a broader range of metabolic benefits, suggesting central adiposity is a more pervasive influence on cardiometabolic health than fitness level in those children [18]. This support the idea that central fat is metabolically detrimental [18].

When considering cardiorespiratory fitness and physical activity levels the relationships with BMI and WHtR are less extensively studied, but existing evidence indicates that higher adiposity is associated with poorer fitness and lower activity [19]. BMI and WHtR both show inverse correlations with fitness and activity. BMI z-scores, WHtR, and shuttle-run fitness performance may predict cardiometabolic risk [19]. This analysis found similar associations for BMI and WHtR with the risk index, and neither index alone was a very strong discriminator [19]. Combining BMI and WHtR together *improved* the identification of at-risk individuals, suggesting that each measure may capture slightly different aspects of risk and that their combination could be informative at least in some subgroups [19].

Research demonstrated that children who accumulated <60 min/day of moderate-to-vigorous physical activity (MVPA) were over twice as likely to be overweight or obese (by BMI criteria) compared to those meeting the activity guideline [20]. Also, MVPA had an independent inverse association with both percentage body fat and WHtR, whereas sedentary time alone showed no significant association with adiposity, however, sedentary behavior did not [20]. It follows that both BMI and WHtR can reflect lifestyle impacts. However, neither BMI nor WHtR alone perfectly identifies all children with elevated cardiometabolic risk, as evidenced by their moderate sensitivities and specificities in most analyses. In summary, the literature suggests that BMI and WHtR are largely congruent indicators of cardiometabolic and functional health in children.

While numerous studies have compared BMI and WHtR in pediatric populations, important gaps remain. First, much of the existing research has focused on either cardiometabolic risk factors or cardiorespiratory fitness. Few studies have concurrently examined a broad spectrum of health indicators, including body composition, metabolic profile, cardiovascular fitness, habitual physical activity, and blood pressure, within the same cohort to directly contrast BMI and WHtR as predictors. Integrating both metabolic outcomes (lipid levels, blood pressure) and functional outcomes (VO₂ₚₑₐₖ, fitness, physical activity, sedentary time) in a single analysis is needed to fully elucidate whether one index consistently outperforms the other across domains.

Second, prior studies often spanning childhood and adolescence together, or target adolescents; less attention has been given specifically to younger children. This age range is important because adiposity assessment may be confounded by pubertal changes in teens, and because early school age is a critical window for prevention.

Third, although WHtR is gaining traction there is ongoing debate about its added value in practice [12]. It may be argued that WHtR could identify high-risk kids that BMI misses, however, concrete evidence is limited. Conversely, others note that BMI performs quite well and that universal WHtR screening might overestimate risk in certain groups, as in short children or those with less linear growth [12].

Finally, there is a lack of consensus on which index is more strongly linked to musculoskeletal outcomes and lean mass in children. BMI could be influenced by muscularity, whereas WHtR is not, but few studies have explicitly examined muscle mass in relation to these indices. These gaps underscore the necessity for targeted research focusing evaluating multiple health dimensions, to determine whether WHtR provides meaningful advantages over the traditional BMI approach.

The present study aims to provide a comprehensive comparison of BMI and WHtR in their ability to predict a range of cardiometabolic and functional health indicators in children aged 6–12 years. This study will address the highlighted gaps by focusing on a narrow pediatric age range and integrating multiple health outcomes.

## Materials and methods

The Health Oriented Pedagogical Project (HOPP), initiated in 2015 in Horten municipality, Norway, was designed to integrate 45 minutes of additional physically active academic instruction, termed active learning, into the daily curriculum of all local primary schools. A comprehensive account of the recruitment strategy and intervention framework has been previously reported [21].

### Study sample

HOPP was conceptualized as a large-scale longitudinal cohort study intended to span from 2015 through 2021, targeting 2816 children aged 6–12 years at baseline. Ultimately, 2297 children were enrolled, representing 82% of the target cohort. Seven primary schools in Horten municipality, South-East in Norway, formed the intervention group (n = 1545), implementing structured active learning, while two schools from the greater Oslo region (n = 752) served as controls. Assessments were conducted in 2015 (grades 1–6) and 2016 (grades 2–7), with declining sample sizes from 2017 onward as pupils transitioned to secondary school. As the COVID-19 pandemic ended data collection prematurely in 2020, this analysis utilizes data from January 15^th^, 2015, to May 15^th^, 2020.

### Anthropometric assessments

Annual measurements were conducted during school hours with children in a non-fasted state. Height was recorded to the nearest 0.1 cm using a SECA 213 stadiometer (SECA GmbH, Hamburg, Germany). Body mass and composition were assessed using a Tanita MC-980MA BIA analyzer (Tanita Corporation, Tokyo, Japan), with a clothing weight correction of 0.4 kg subtracted from the measured body mass. BMI was computed as weight divided by height squared (kg/m²). Waist circumference was taken at the umbilical level following normal expiration, using a non-elastic anthropometric tape, and recorded to the nearest 0.5 cm. WHtR was calculated by dividing waist circumference by height (both in cm). Measurements of body fat percentage and muscle mass derived from bioimpedance analysis were included to evaluate how well WHtR and BMI approximate advanced anthropometric health-risk indicators.

### Fitness

Endurance was assessed using the Andersen intermittent running test [22]. The test was conducted in school gymnasiums and consisted of alternating 15 s running and 15 s rest for a total of 10 minutes. Performance was expressed as total distance covered, calculated from the number of completed laps [22].

Direct measurement of VO₂peak was restricted to 1st-grade pupils in 2015. The same cohort was subsequently tested annually until the HOPP project ended in 2020. An incremental treadmill protocol was applied, starting at 5 km·h ⁻ ¹ with a 5% incline for 5 minutes, followed by an increase to 7 km·h ⁻ ¹. Speed was then increased by 1 km·h ⁻ ¹ per minute until 10 km·h ⁻ ¹ was reached. Thereafter, treadmill incline was increased by 1% per minute until volitional exhaustion [23]. In 2015, oxygen uptake was recorded using a K4b2 metabolic system (Cosmed Srl, Rome, Italy) with breath-by-breath data averaged over 30-s intervals. From 2016 onward, a K5 metabolic analyzer with mixing-chamber measurements was used. Heart rate during the test, the highest recorded heart rate (HRpeak), and time to exhaustion (TTE) were recorded.

### Physical activity

Physical activity (PA) was measured using ActiGraph wGT3X-BT accelerometers (ActiGraph LLC, Pensacola, FL, USA). Children were instructed to wear the device on the right hip using an elastic belt for seven consecutive days, removing it only when injured, ill, absent from school, showering, or swimming. Devices were set to a sampling frequency of 100 Hz, aggregated to 10-s epochs. For inclusion in analyses, participants were required to have at least 8 hours/day of recorded wear time.

Non-wear time was identified and removed using the Troiano algorithm, defined as ≥60 minutes of consecutive zero counts, allowing 2 minutes of activity tolerance [24]. Valid wear time was defined as 06:00–23:59. Time spent in intensity categories was derived from mean counts per minute (cpm): sedentary (0–99 cpm), light (100–1999 cpm), moderate (2000–4999 cpm), and vigorous (≥5000 cpm), with minutes accumulated in each intensity domain [24]. Moderate-to-vigorous physical activity (MVPA) was computed as the sum of minutes spent in the moderate and vigorous domains.

### Blood pressure

Blood pressure (BP) was measured seated with an automated monitor (Omron M6 Comfort IT, HEM-7322U-E) using an Intelli Wrap cuff (HEM-FL31) adjusted for children on the left upper arm. Up to three readings were obtained and repeat measurements were taken if the device failed to register or if values were outside age-adjusted reference ranges. If three attempts were unsuccessful, BP was measured on the right arm until a valid reading was obtained.

### Blood samples

Not all children/parents consented to blood sampling; therefore, 1,344 samples were available at baseline (2015) for the hematological parameters included in this study, and 1,185 samples were collected in 2016. From 2017 to 2020, blood sampling was restricted to 7th-grade pupils. Samples were obtained non-fasting between 08:00 and 13:30, and participants were instructed to avoid strenuous exercise prior to sampling. Trained phlebotomists collected venous blood from the antecubital vein into 4 mL K2EDTA tubes (Vacuette®, Greiner Bio-One, Austria). Sampling was performed at the schools, and analyses were conducted using standard procedures at the central laboratory at Vestfold Hospital Trust (accredited according to NS-EN ISO 15189). All analytes were individually accredited and participated in external quality assurance through NOKLUS [25]. Hematology was analyzed on a Sysmex XE-2100 (Sysmex Corporation, Kobe, Japan) using manufacturer reagents.

### Statistical analyses

Descriptive statistics were used to summarize participant characteristics and study variables across measurement years. The distributions were inspected, and reasonably symmetry were found, and linear mixed-model assumptions were considered acceptable. Pearson correlation analyses were used to screen for multicollinearity; because average and total MVPA were highly correlated (r = 0.81), average MVPA was retained for subsequent analyses.

To account for repeated measurements, we fitted linear mixed-effects models (LMMs) with a random intercept for each child (participant ID). Models were estimated using restricted maximum likelihood (REML), with study year treated as the repeated time factor and a diagonal residual covariance structure to allow heterogeneous residual variances across time points. Each health measure was analyzed as a separate outcome in its own model. In each model, the main exposure was either BMI or WHtR (fitted in separate models), with age (continuous) and sex included as core covariates. Models were additionally adjusted for the following health-related covariates: physical activity (average MVPA and sedentary time), cardiorespiratory fitness (VO₂peak and Andersen test distance), blood pressure (systolic and diastolic), lipid profile (total cholesterol, HDL, non-HDL), and body composition (fat percentage and muscle mass). Least squares mean (adjusted means) were estimated at the sample mean of the covariates, and regression estimates were interpreted as associations with the outcomes.

LMMs were performed using NCSS 2024 (v24.0.1), and other analyses in IBM SPSS Statistics for Windows, Version 28.0 (IBM Corp., Armonk, NY, USA). Statistical significance was set at α = 0.05; results are presented with standardized deviations and p-values.

Attrition across study years occurred primarily because pupils transitioned from primary to secondary school, resulting in fewer available annual school-based measurements (approximately 350 children per year). We summarized the number of observations per year and the distribution of repeated measurements per child. To evaluate the plausibility of the missing-at-random assumption for the mixed-effects models, baseline (2015) characteristics were compared between children with limited follow-up (1–2 measurements) and those with more complete follow-up (≥3 measurements) using appropriate tests for continuous and categorical variables. In addition, we modelled follow-up completeness as a function of baseline characteristics to examine whether attrition was associated with observed covariates.

Because multiple outcomes across several domains were examined, the analyzes should be interpreted with some caution regarding multiplicity, and findings from secondary outcome analyzes should be considered exploratory rather than strictly confirmatory.

### Ethics and consent

This study adheres to the ethical standards outlined in the Declaration of Helsinki. The research protocol was approved by the Regional Committee for Medical Research Ethics (REK South-East, reference no. 2014/2064/REK) and registered at ClinicalTrials.gov (Identifier: NCT02495714; registration date: June 20, 2015). All personal identifiers were replaced with unique ID codes, and data were securely stored after each annual test cycle. During statistical analysis, only anonymized data were used. Written informed consent was obtained from the legal guardians of all participating children.

## Results

### Body composition

Descriptive statistics for anthropometric measures from 2015 to 2020 are presented in Table 1. Overall, weight and height increased progressively each year, reflecting normal growth patterns. WHtR and BMI showed relatively stable trends over time, with minor fluctuations from year to year. Standard deviations remained consistent across years, indicating stable variability within the sample throughout the study period.

**Table 1:** Descriptive statistics for weight, height, waist-to-height ratio (WHtR), and body mass index (BMI) from 2015 (baseline) to 2020.

		Boys				Girls			
		Weight (kg)	Height (cm)	WHtR	BMI	Weight (kg)	Height (cm)	WHtR	BMI
**2015**	**n**	1150	1150	1099	1150	1121	1122	1080	1121
	**Mean**	33,4	138,7	0,45	17,07	33,3	138,3	0,45	17,04
	**SD**	9,22	11,49	0,04	2,64	9,90	12,05	0,05	2,94
**2016**	**n**	1075	1085	1071	1071	1041	1049	1042	1038
	**Mean**	38,4	144,5	0,45	18,05	38,2	144,0	0,44	18,06
	**SD**	10,56	11,64	0,05	2,88	11,00	12,17	0,05	3,05
**2017**	**n**	823	837	829	822	785	810	795	786
	**Mean**	39,1	146,4	0,45	17,95	39,2	146,2	0,45	18,08
	**SD**	10,28	10,38	0,05	2,96	10,58	10,71	0,05	3,19
**2018**	**n**	609	613	592	607	586	590	571	587
	**Mean**	41,2	149,0	0,46	18,37	41,4	149,0	0,45	18,50
	**SD**	10,28	9,36	0,05	3,03	10,72	9,74	0,05	3,38
**2019**	**n**	445	445	434	444	401	402	388	402
	**Mean**	43,3	152,4	0,45	18,42	43,6	152,3	0,45	18,71
	**SD**	10,17	9,18	0,05	2,94	9,82	8,53	0,05	3,34
**2020**	**n**	282	281	271	278	261	258	247	256
	**Mean**	45,8	154,8	0,45	18,90	46,6	154,9	0,44	19,31
	**SD**	10,58	8,52	0,05	3,16	10,56	8,17	0,06	3,50

Waist-to-height ratio (WHtR), Body Mass Index (BMI), Standard Deviation (SD), N = number of measurements.

In the mixed models (adjusted for sex and age) (Table 2), higher BMI and WHtR were each significantly associated with greater percent body fat (both p < 0.001). Using standardized coefficients, BMI showed a stronger association with percent fat than WHtR (BMI β = 0.78 vs WHtR β = 0.46). Both measures also captured sex differences, with boys having significantly lower percent fat than girls (B = –3.5, p < 0.001).

**Table 2:** Linear mixed model estimates (β standardized coefficients with p-values) for BMI and WHtR in relation to each health outcome (separate models).

	BMI β	WHtR β
**Body composition**		
Fat (%)	0.78, p < .001	0.46, p < .001
Muscle mass (kg)	0.42, p < .001	0.11, p < .001
**Blood lipids**		
Total cholesterol (mmol/L)	0.05, p = 0.15	0.06, p < .001
HDL (mmol/L)	–0.25, p < .001	–0.18, p < .001
Non-HDL (mmol/L)	0.18, p < .001	0.15, p < .001
**Cardiorespiratory fitness**		
VO₂peak (mL⦁kg^-1^⦁min^-1^)	–0.54, p < .001	–0.31, p < .001
Andersen test (m)	–0.28, p < .001	–0.24, p < .001
**Physical activity**		
MVPA (min/day)	0.01, p < .001	0.02, p < .001
Sedentary (min/day)	–0.04, p = .79	–0.03, p = .58
**Blood pressure**		
Systolic BP (mmHg)	0.18, p < .001	0.01, p < .001
Diastolic BP (mmHg)	0.08, p < .001	0.08, p < .001

Body Mass Index = BMI, Waist-to-height ratio = WHtR, High-Density Lipoprotein = HDL, non-High-Density Lipoprotein = NonHDL, Peak oxygen uptake = VO_2_peak, Andersen intermittent running test = Andersen test, Moderate-to-Vigorous Physical Activity = MVPA, Sedentary behavior = Sedentary, Blood Pressure = BP.

In the mixed models (adjusted for sex and age) (Table 2), higher BMI and WHtR were each significantly associated with greater percent body fat (both p < 0.001). Using standardized coefficients, BMI showed a stronger association with percent fat than WHtR (BMI β = 0.78 vs WHtR β = 0.46). Both measures also captured sex differences, with boys having significantly lower percent fat than girls (B = –3.5, p < 0.001).

### Lipids

Across lipid outcomes, both BMI and WHtR were significantly associated with less favourable lipid profiles, with differences in magnitude generally modest (Table 2). For total cholesterol, WHtR showed a small but significant positive association (β = 0.06, p < 0.001), whereas the association with BMI was not statistically significant (β = 0.05, p = 0.15). For HDL, higher adiposity was associated with lower (less favourable) HDL for both indices, with a stronger inverse association for BMI than for WHtR (BMI β = –0.25 vs WHtR β = –0.18; both p < 0.001). Similarly, non-HDL cholesterol was positively associated with both indices, again with a slightly stronger association for BMI than for WHtR (BMI β = 0.18 vs WHtR β = 0.15: both p < 0.001). Overall, WHtR was uniquely associated with total cholesterol in this cohort, whereas BMI showed slightly stronger associations with HDL and non-HDL cholesterol.

Higher adiposity was associated with lower cardiorespiratory fitness, with both BMI and WHtR showing inverse associations with VO₂peak and running performance. In mixed models adjusted for age and sex (Table 2), VO₂peak was inversely associated with both indices, with a stronger standardized association for BMI than for WHtR (BMI β = –0.54 vs WHtR β = –0.31; both p < 0.001). The Andersen intermittent running test showed a similar pattern, with inverse associations for both measures and comparable magnitudes (BMI β = –0.28 vs WHtR β = –0.24; both p < 0.001). Boys demonstrated higher VO₂peak and longer Andersen distances than girls.

### Physical activity

In Table 2, BMI and WHtR showed statistically significant but very small associations with MVPA (BMI β = 0.01; WHtR β = 0.02; both p < 0.001), indicating minimal differences in daily MVPA across adiposity levels. Boys accumulated more MVPA than girls, and MVPA declined with age. In contrast, neither BMI nor WHtR was significantly associated with sedentary time (BMI β = –0.04, p = 0.79; WHtR β = –0.03, p = 0.58).

### Blood pressure

Both BMI and WHtR were positively associated with systolic and diastolic blood pressure (Table 2). However, standardized coefficients indicated a markedly stronger association between BMI and systolic BP compared with WHtR (BMI β = 0.18 vs WHtR β = 0.01; both p < 0.001), whereas associations with diastolic BP were similar for BMI and WHtR (both β = 0.08; p < 0.001). Overall, the standardized associations were small in magnitude, indicating limited explained variance in blood pressure by either anthropometric index.

Age was strongly associated with BP, with each year associated with a 1.5 mmHg increase in SBP and a 0.61 mmHg decrease in DBP. Overall, boys had higher SBP but marginally lower DBP than girls. After accounting for age and sex, both higher BMI and WHtR remained significantly associated with increased SBP and DBP.

**Fig 1:**
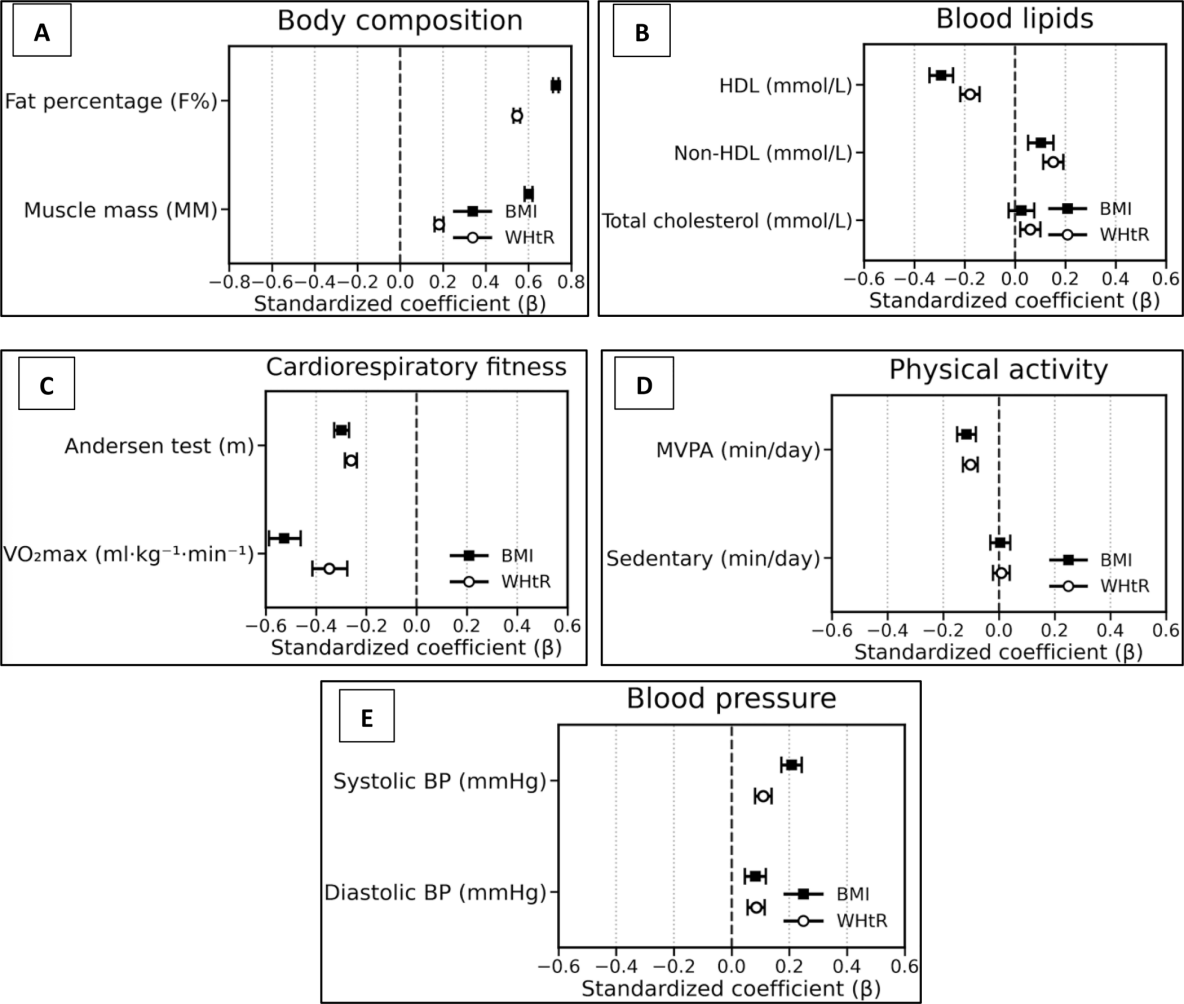
Forest plots comparing the strength of associations between BMI vs. WHtR and each outcome. Each panel corresponds to a domain: (a) body composition, (b) blood lipids, (c) cardiorespiratory fitness, (d) physical activity, and € blood pressure. Blue squares represent the regression coefficient (β) for BMI and red circles for WHtR, with horizontal lines indicating the 95% confidence intervals. The vertical dashed line at β = 0 represents no association.

In general, WHtR showed associations of equal or greater magnitude than BMI for most health variables examined. This was especially evident for lipid profiles. Table 2 presents a comparison of the regression coefficients for BMI and WHtR across all outcomes. Fig 1 illustrates that WHtR often has a larger magnitude of association (i.e., coefficients further from zero) than BMI. WHtR lies further from zero in panels (b) and (c), indicating stronger associations with lipid and fitness outcomes, whereas for body composition and blood pressure (panels a and e) BMI and WHtR are closer together, suggesting both predictors perform similarly.

Fig 1 summarizes the standardized coefficients (β) for BMI and WHtR across outcome domains, with the vertical dashed line indicating no association. In panel (a) body composition, BMI lies further from zero than WHtR for both fat percentage and muscle mass, indicating stronger associations for BMI. In panel (b) blood lipids, the pattern is mixed: WHtR shows a small positive association with total cholesterol while BMI is close to zero and not statistically significant, whereas BMI shows slightly stronger associations than WHtR for HDL (more negative) and for non-HDL/LDL (more positive). In panel (c) cardiorespiratory fitness, BMI shows a clearly stronger inverse association with VO₂peak than WHtR, while the associations with Andersen running performance are similar in magnitude. In panel (d) physical activity, coefficients for both BMI and WHtR cluster close to zero for MVPA and sedentary time, indicating very small associations overall. In panel (e) blood pressure, BMI shows a markedly stronger association with systolic blood pressure than WHtR, whereas diastolic blood pressure associations are similar for the two indices.

Using age- and sex-specific IOTF cut-offs for BMI classification, cross-tabulation with WHtR showed partial disagreement between the two measures (Table 3). Across all test years combined, 351 of 6897 children classified as non-overweight by BMI (5.1%) had WHtR ≥ 0.5. Conversely, 351 of 1305 children with WHtR ≥ 0.5 (26.9%) were not classified as overweight/obese by BMI. The same overall pattern was observed in each test year. The proportion of children classified as non-overweight by BMI but with WHtR ≥ 0.5 ranged from 3.5% in 2016 to 7.6% in 2019.

**Table 3:** BMI categories were derived using age- and sex-specific IOTF cut-offs and collapsed into non-overweight and overweight/obese. WHtR was classified as <0.5 or ≥0.5.

BMI category	WHtR < 0.5, n (%)	WHtR ≥ 0.5, n (%)	Total, n
**Non-overweight**	6546 (94.9%)	351 (5.1%)	6897
**Overweight/obese**	410 (30.1%)	954 (69.9%)	1364
**Total**	6956	1305	8261

## Discussion

Both BMI and WHtR were significantly associated with multiple health indicators in children, including adverse blood lipid profiles, poorer aerobic fitness, and endurance. The standardized results indicate that BMI shows equal or stronger associations than WHtR across most outcomes, with the most pronounced differences for body composition, VO₂peak, and systolic blood pressure. For lipids, findings are outcome specific as WHtR shows the clearer association with total cholesterol, whereas BMI shows slightly stronger associations with HDL and non-HDL/LDL. Associations with endurance and diastolic blood pressure are broadly comparable between BMI and WHtR, while associations with physical activity are small in magnitude. Overall, differences between WHtR and BMI in the magnitude of associations were modest. Importantly, these results are derived from annual repeated measures within a narrow age range (6–12 years) and across both metabolic and functional outcomes, allowing evaluation of consistent within-child associations during late childhood.

### Previous findings

Prior studies generally report that BMI and WHtR perform similarly for cardiometabolic risk classification in pediatric populations, with modest discrimination (e.g., AUCs typically <0.75) [16,26]. Consistent with this, we observed broadly comparable associations for BMI and WHtR across outcomes [16,27].

However, some evidence suggests WHtR may better capture certain risks, especially related to central fat distribution, which is metabolically harmful. Some studies suggest WHtR may better reflect central adiposity and adiposity-related outcomes than BMI [28]. Our data showed only a slight WHtR advantage in its association with percent fat, but the trend supports the notion that central obesity indices can be more sensitive to adiposity-related outcomes. Indeed, WHtR has been linked to cardiometabolic risk even among children with “normal” BMI [4,26]. Ashwell and colleagues have highlighted that relying on BMI alone can miss many at-risk individuals; in the UK, approximately 10% of the total population have a high WHtR and would be overlooked if only BMI were considered [5].

### Present findings

The present study revealed that standardized coefficients showed that BMI had stronger associations than WHtR with both percent body fat and muscle mass, consistent with BMI more strongly reflecting overall body size and general adiposity in this age group. WHtR remained significantly associated with fat percentage, supporting its relevance as a marker of central adiposity despite a smaller association magnitude than BMI for these body-composition outcomes.

BMI showed slightly stronger associations than WHtR with HDL and non-HDL cholesterol, while WHtR was the only index significantly associated with total cholesterol. These differences were small in magnitude, supporting the interpretation that BMI and WHtR provide broadly comparable information for lipid-related risk in late childhood, with potential complementary value depending on the lipid outcome considered.

A clear inverse association between adiposity and cardiorespiratory fitness was revealed. BMI exhibited a stronger association with VO₂peak than WHtR (β = –0.54 vs –0.31), suggesting that overall adiposity to aerobic capacity is better captured by BMI in this age group. For the Andersen test, BMI and WHtR showed very similar inverse associations, indicating broadly comparable relationships.

Associations between adiposity indices and accelerometry-derived MVPA were statistically significant but small in magnitude, suggesting that differences in habitual MVPA across adiposity levels were modest in this cohort. Neither BMI nor WHtR was associated with sedentary time, indicating that higher adiposity was not simply a function of accumulating more sedentary minutes when accounting for age and sex.

Standardized coefficients suggested that BMI was more strongly associated with systolic blood pressure than WHtR, whereas associations with diastolic blood pressure were comparable. Overall, the standardized magnitudes were small, indicating that adiposity indices explain only a limited proportion of blood pressure variation in this age group. Although standardized effect sizes for blood pressure outcomes were numerically small, translating these associations into realistic contrasts (e.g., 10th–90th percentile differences in BMI or WHtR) indicates systolic blood pressure differences of approximately 2–4 mmHg. Such differences are modest at an individual level but may be clinically meaningful at a population level, particularly given the early age of exposure and the tendency for blood pressure to track over time.

The present study observed cases of children with a WHtR ≥ 0.5 who were not classified as overweight based on BMI (Table 3), as observed by others [8,26]. This suggests WHtR may *add* value by flagging centrally obese, shorter-stature children who have disproportionate abdominal fat but might be average in weight for height. Such children may have less favorable metabolic profiles despite a non-overweight BMI. We also noted instances of high-BMI children with only moderate WHtR, indicating a more peripheral distribution of weight; these children may have slightly better metabolic profiles than their BMI alone would suggest. Taken together, our findings and prior research indicate that BMI and WHtR generally concur in risk assessment, but each can uniquely identify a subset of children that the other might misclassify.

The results on blood pressure and fitness add nuance to the BMI–WHtR comparison as WHtR was slightly stronger associated with elevated blood pressure than BMI. This echoes studies linking central obesity to higher systolic BP in youth [28]. Meanwhile, the inverse association between adiposity and aerobic fitness was similarly captured by both BMI and WHtR in our analysis. Notably, few pediatric studies have examined these anthropometric indices against objectively measured fitness and accelerometry-derived activity within a repeated-measures framework in this age band. The present study demonstrated similarly that both high BMI and WHtR were significantly associated with poor fitness and activity levels. This indicating that general adiposity is the primary factor in reduced childhood fitness**,** with central fat playing a secondary role.

Overall, our study corroborates the consensus that BMI and WHtR are both useful but imperfect proxies for health risks in children. Neither index captures metabolic risk with high sensitivity, as many children with adverse biomarkers do not exceed conventional BMI/WHtR cutoffs. In our cohort, anthropometric cut-offs identified children with elevated risk markers with few false positives, but some children with dyslipidaemia or higher blood pressure remained below conventional thresholds. Our data reflect this trade-off as well; most children flagged by BMI/WHtR truly had risk factor elevations, with few false positives. However, many children with dyslipidemia or prehypertension fell below overweight thresholds, showing false negatives. This underscores that anthropometric screening alone cannot identify all high-risk children, a limitation previously documented [16]. This highlights the continued value of complementary assessment, like lipids and fitness testing, beyond anthropometrics alone.

### Practical implications

From a practical standpoint, WHtR offers some notable advantages over BMI for routine screening. Both metrics are inexpensive and non-invasive to obtain, requiring only a scale and stadiometer for BMI, and in addition, a simple tape measure for WHtR. However, interpreting BMI in children is not straightforward, as it requires age- and sex-specific growth charts or z-score calculators to determine whether a child’s BMI is “high” for their age [29].

WHtR, by contrast, has a single universal threshold **(**≥0.5) proposed for both children (≥6 years old) and adults, which can be easily calculated and immediately interpreted without reference Tables (5). This ease of use is reflected in public health messaging like **“**Keep your waist to less than half your height,**”** which is intuitive for all. Our findings suggest that employing such a WHtR cutoff would identify nearly all of the high-BMI children flagged by conventional criteria, *plus* some additional children with normal-weight BMI but with central obesity. Thus, WHtR could be a more sensitive initial screen for central adiposity-related risk. Indeed, the UK’s National Institute for Health and Care Excellence (NICE) has recently incorporated WHtR into pediatric obesity assessment and advises using WHtR in conjunction with BMI in children [12].

BMI remains the more established tool, with well-known percentile thresholds [30]. Clinicians and growth charts worldwide have traditionally relied on BMI cut points to trigger further evaluation. We do not advocate abandoning BMI; rather, our results support integrating WHtR as a complementary metric. In a short child with excess abdominal fat, WHtR may signal risk even if BMI is below the overweight cutoff. Conversely, in a tall, muscular preteen with borderline high BMI, a normal WHtR could reassure that central adiposity is not high. In practice, measuring waist circumference adds minimal exam time and provides actionable information. In screening programs, a practical approach might be to use WHtR ≥ 0.5 as an initial universal cut-point to flag central obesity, and then follow up with BMI percentiles and clinical evaluations for those children to obtain a complete picture of health risks.

### Strengths and limitations

By following children through late childhood with annual assessments, we captured within-person trajectories and used linear mixed models to strengthen the analysis. We also assessed a broad range of health indicators, from laboratory biomarkers to cardiorespiratory fitness and objectively measured activity, providing a comprehensive picture of cardiometabolic health beyond the typical focus on blood pressure and lipids. Notably, the inclusion of standard fitness measures and accelerometry-based physical activity tracking is a strength, as few studies have objectively linked anthropometrics with functional fitness and daily activity in children. The use of validated protocols and equipment for body composition, blood assays, and blood pressure adds to the rigor. Key strengths of this study also include the large, well-characterized cohort with repeated measures over time. This approach increases power and accounts for growth-related changes, yielding more robust estimates of BMI/WHtR–health outcome relationships than a purely cross-sectional design. Additionally, directly comparing BMI and WHtR within the same statistical models allowed us to contrast the magnitude of their associations under identical conditions, which bolsters the internal validity of the comparison.

We acknowledge several limitations. This study evaluates associations and does not quantify predictive performance; therefore, conclusions are limited to comparative associations rather than prediction. First, the sample is regionally based, which may limit generalizability. Cultural, ethnic, and genetic factors influence body fat distribution; thus, our findings might not be directly applicable to populations with different growth patterns. The children in our study were predominantly ethnically Norwegian and relatively homogeneous, and the prevalence of overweight individuals was low, which could affect the strength of the observed associations. Second, while WHtR is conceptually simple, waist circumference measurement in children may be variable**.** We used standard protocols, but differences in measurement technique or child posture can introduce error. On the other hand, BMI, although simpler to measure, has the disadvantage of not distinguish body composition. Finally, our study’s observational design precludes conclusions about causality. While excess adiposity likely contributes to poorer health outcomes, it is also plausible that children with underlying metabolic issues gain more abdominal fat. Long-term longitudinal studies of adolescence would be valuable for confirming the directionality and persistence of these relationships.

A limitation is that multiple outcomes were tested across several domains, which may have increased the risk of type I error. Hence, some statistically significant findings may represent chance findings and should therefore be interpreted cautiously, particularly for secondary outcomes.

## Conclusion

In this cohort of children aged 6–12 years followed with annual repeated measurements, the findings of BMI and WHtR suggested associations with a range of cardiometabolic and functional health indicators, implying their utility as simple anthropometric markers in pediatric health assessment. Using standardized coefficients, BMI indicate equal or stronger associations than WHtR for most outcomes, while WHtR suggested complementary information by better reflecting central fat distribution. A key practical advantage of WHtR is its single, universal cut-off (≥0.5) that can be applied across ages and both sexes, making it easier to interpret than BMI, which requires age- and sex-specific reference standards. Accordingly, WHtR should not be viewed as a substitute for BMI, but rather as a pragmatic addition that may help identify children with disproportionate abdominal adiposity who may not be flagged by BMI-based criteria alone. For clinical practice and surveillance, incorporating WHtR alongside BMI may therefore improve the interpretability of screening and provide a more complete picture of adiposity-related risk. Future studies may confirm these findings in more diverse populations and evaluate whether WHtR adds independent value beyond BMI for longer-term trajectories of cardiometabolic risk.
